# Leptin and adiponectin as predictors of cardiovascular risk after gestational diabetes mellitus

**DOI:** 10.1186/s12933-016-0492-4

**Published:** 2017-01-10

**Authors:** Tove Lekva, Annika Elisabet Michelsen, Pål Aukrust, Tore Henriksen, Jens Bollerslev, Thor Ueland

**Affiliations:** 1 Research Institute of Internal Medicine, Oslo University Hospital, Rikshospitalet, Oslo, Norway; 2 Section of Clinical Immunology and Infectious Diseases, Oslo University Hospital, Rikshospitalet, Oslo, Norway; 3 Department of Obstetrics, Oslo University Hospital, Rikshospitalet, Oslo, Norway; 4 Section of Specialized Endocrinology, Department of Endocrinology, Oslo University Hospital, Rikshospitalet, Oslo, Norway; 5 Faculty of Medicine, University of Oslo, Oslo, Norway; 6 K. G. Jebsen Thrombosis Research and Expertise Center, University of Tromsø, Tromsø, Norway

**Keywords:** GDM, CVD, Leptin, Adiponectin

## Abstract

**Background:**

Gestational diabetes mellitus (GDM) is a significant risk factor for cardiovascular disease (CVD) in later life, but the mechanism remains unclear. Adipokine imbalance in the presence of metabolic dysfunction may be a key event in promoting CVD. The aim of the study was to examine the relationships between GDM, cardiovascular risk, and plasma adiponectin, leptin and the leptin/adiponectin (L/A) ratio in pregnancy and at 5 years after the index pregnancy.

**Methods:**

This population-based prospective cohort included 300 women who had an oral glucose tolerance test (OGTT) during pregnancy. Five years later, the OGTT was repeated along with dual-energy X-ray absorptiometry, lipid analysis, and pulse wave velocity analysis. Fasting adiponectin and leptin levels were measured four times during pregnancy and at follow-up.

**Results:**

We found the L/A ratio higher in GDM women both during pregnancy and follow-up compared to non-GDM women. A high L/A ratio during pregnancy was associated with CV risk based on lipid ratios at follow-up, especially the TG/HDL-C ratio. Further, interaction analysis indicated that an increase in the L/A ratio of 1 unit was associated with a higher CV risk in GDM compared to normal pregnancy. Finally, low adiponectin levels independently predicted increased lipid ratios at follow-up.

**Conclusions:**

Taken together, our findings suggest that high L/A ratio in pregnancy and in particularly in those with GDM are associated with an unfavorable CVD risk profile during follow-up. Future studies should investigate if a dysregulated leptin and adiponectin profile during pregnancy is associated with atherosclerotic disease during long-term follow-up.

**Electronic supplementary material:**

The online version of this article (doi:10.1186/s12933-016-0492-4) contains supplementary material, which is available to authorized users.

## Background

Adiponectin and leptin are two adipocytokines or adipokines that have been studied extensively due to their association with insulin resistance, obesity and cardiovascular (CV) risk. Secretion of leptin influence body weight, positively associated with percentage body fat, suggesting that leptin levels is mediating adiposity signals to the brain [1]. Excessive production of leptin is a consequence of resistance to its effect on target organs [2], and increased levels are associated with high BMI and insulin resistance in type 2 diabetes patients (T2DM) [3]. In contrast to leptin, adiponectin has antidiabetic properties, and these hormones have also opposing effects on subclinical inflammation. Whereas leptin up-regulates TNF and interleukin (IL)-6, adiponectin down-regulates these and several other inflammatory mediators [4].

The possibility that leptin and adiponectin may also be relevant to vascular disease has been suggested by experimental studies showing that leptin promotes atherosclerosis and thrombosis in apolipoprotein E (ApoE)-deficient mice [5], whereas adiponectin has been shown to attenuate atherosclerosis in mice prone to develop atherosclerosis [6]. Furthermore, numerous clinical studies implicate dysregulated leptin and adiponectin levels in the progression of T2DM, coronary artery disease and hypertension [7, 8]. Also, in individuals without manifest CV disease (CVD), high circulating leptin and low adiponectin levels are associated with multiple CVD risk including lipid dysregulation [1]. Dyslipidemia is a major risk factor for CVD and lipid ratios have been shown to predict CVD risk better than the individual lipids used independently [9].

Due to the opposite metabolic effects of leptin and adiponectin, the leptin/adiponectin ratio (L/A ratio) has been proposed as a useful marker for metabolic disease [10, 11] and may be more strongly associated with T2DM risk and first CV event than leptin and adiponectin alone [12, 13]. A normal pregnancy results in increased insulin resistance. Adiponectin decreases from mid pregnancy [14] and leptin increases throughout pregnancy [15]. Although not consistently demonstrated, GDM women have lower adiponectin and higher leptin levels compared to non-GDM women [16, 17] and the L/A ratio has been associated with insulin resistance (HOMA-IR) during pregnancy [18].

Leptin, adiponectin and the L/A ratio levels have not been prospectively evaluated during pregnancy and related to future maternal CVD risk. GDM women are more prone to develop CVD later in life and our overarching hypothesis is that this enhanced risk may start to develop or accelerate during pregnancy. We measured circulating leptin and adiponectin in 300 women from a prospective cohort study at multiple times during pregnancy and at 5-year follow-up. We hypothesized that the L/A ratio during pregnancy and at follow-up is associated with GDM and with CV risk at 5 years as evaluated by unfavorable lipid ratios.

## Methods

### Study population

The STORK study was a prospective cohort study with a longitudinal design in which 1031 low-risk women of Scandinavian heritage who gave birth at Oslo University Hospital Rikshospitalet between 2002 and 2008 were followed throughout their pregnancy as previously described [19]. Exclusion criteria included multiple pregnancy, known pre-gestational diabetes, severe chronic medical conditions (such as lung, cardiac, gastrointestinal or renal diseases), and pregnancies complicated by major fetal malformations. Briefly, each pregnant woman had four antenatal visits at gestational age (GA) weeks 14–16, 22–24, 30–32, and 36–38. Clinical data and blood samples were collected at each visit, processed, and stored at −80 °C until further analysis. A 75 g oral glucose tolerance test (OGTT) was performed on all women at antenatal GA visit 14–16 and 30–32 weeks.

The current study is a 5-year follow-up after the index pregnancy [20]. A total of 1031 participants from the original STORK cohort were invited to participate. Exclusion criteria included pregnancy at the time of invitation and/or delivery within the past year. Three hundred women agreed to participate. At the time of the 5-year follow-up visit, a fasting blood draw was performed to measure lipid profiles and a 75 g OGTT was conducted. For the purposes of this analysis, the term primiparous is used to identify women delivering their first child in the index pregnancy (nulliparous) or with only one prior delivery at 5-year follow-up. Women with preeclampsia, preterm birth and hypertension without GDM were excluded in this particular study.

### Measurements of glycemic and lipid parameters

All 75 g OGTTs were performed in the morning after an overnight fast. Venous EDTA blood was analyzed at point of care using an Accu-Check Sensor glucometer (Roche Diagnostics GmbH, Mannheim, Germany). Additional venous blood samples were allowed to clot for 30 min and the serum separated by centrifugation for 10 min at 3000*g* and stored at −80 °C. Glucose levels were also measured from frozen serum samples collected at 30–32 weeks using the hexokinase method (Hitachi Modular P800, Roche Diagnostics, Mannheim, Germany) at an accredited clinical chemistry laboratory at Oslo University Hospital Rikshospitalet, as previously reported [20]. For the 5-year follow-up study, we used the glucose data from the Accu-check Sensor glucometer (Roche Diagnostics, Mannheim, Germany). Insulin levels in the stored samples were assayed in duplicate by RIA (Diagnostic Products Corporation, Los Angeles, CA, USA), as previously reported [20]. Levels of apolipoprotein A (apoA), apoB, HDL-C, low density lipoprotein cholesterol (LDL-C) (direct measurements), and triglycerides (TG) were measured from frozen serum samples at follow-up at the clinical chemistry laboratory at Oslo University Hospital Rikshospitalet. The ratios of TG/HDL-C, LDL/HDL-C and apoB/apoA are known risk factors for CVD [9, 21, 22], and were calculated based on the above measurement. For leptin and adiponectin analysis, we used fasting plasma from venous EDTA blood sampled on ice, centrifuged for 25 min at 3000*g* at 4 °C, separated, and stored at −80 °C until analyzed. Total adiponectin and leptin were measured in duplicate using a commercially available enzyme-linked immunosorbent assay (ELISA; R & D Systems, Minneapolis, MN, USA) in a 384 format using the combination of a SELMA (Jena, Germany) pipetting robot and a BioTek (Winooski, VT, USA) dispenser/washer (EL406). Absorption was read at 450 nm with wavelength correction set to 540 nm using an ELISA plate reader (Synergy H1 Hybrid, Biotek, Vinooski, VT, USA).

### Diagnosis of GDM

GDM was diagnosed on a 75 g OGTT using the WHO criteria from 1999: 2 h plasma glucose ≥7.8 mmol/L [23]. Estimation of insulin sensitivity was measured on the same samples collected at the time of OGTT using the Matsuda index (i.e., 10,000/square root of [fasting glucose (mmol/L) × fasting insulin (mU/L)] × [mean glucose (mmol/L) × mean insulin (mU/L)]) during OGTT. This index is a measure of whole body insulin sensitivity that has been validated against the euglycemic-hyperinsulinemic clamp [24]. Estimation of β-cell function was assessed with the insulin secretion-sensitivity index (ISSI-2) (area under the curve (AUC) insulin (mU/L)_0–120_/glucose (mmol/L)_0–120_ × Matsuda), which has been validated against the disposition index from the intravenous GTT [25]. Estimation of homeostasis model assessment: insulin resistance (HOMA–IR) was calculated as fasting insulin (mU/L) × fasting glucose (mmol/L)/22.5, as previously described by Matthews et al. [26]. Although the insulin sensitivity has been validated against the euglycemic-hyperinsulinemic clamp and the β-cell function against the disposition index from the intravenous GTT, these are estimates, and therefore a limitation of the study.

### Measurements of arterial stiffness

Aortic stiffness was assessed by means of PWV measurements using SphygmoCor (Atcor Medical, Sydney, Australia), a non-invasive technique with direct-contact pulse sensors, and the method and results have previously been published [27].

### Measurements of body fat composition

Total body composition was determined by dual-energy X-ray absorptiometry (DXA; GE Lunar Prodigy Densitometer, software version 12.10, GE Medical Systems, Lunar Corp., Madison, WI, USA) and analyzed using enCORE software (version 14.10; GE Medical Systems), as previously described [20].

### Statistical analysis

Statistical analyses were conducted using SPSS for Windows, version 21.0 (Chicago, IL, USA). Data are expressed as mean ± SD when normally distributed and median (25th, 75th percentile) when skewed. Comparison between women with and without a history of GDM was performed using *t* test or Mann–Whitney U depending on distribution, and Chi square test for categorical variables. Several multiple regression models were used in the study. We first performed a stepwise linear regression to determine the most important and consistent predictors of the L/A ratio at multiple times during pregnancy (listed in Table 2). These were used as adjustment variables in subsequent forced logistic regression analysis when calculating odds ratios for leptin, adiponectin and the L/A ratio according to established cut-offs for the apoB/apoA, LDL/HDL-C ratio and TG/HDL-C ratio. We also performed linear regression to identify the strongest predictors at 5 years follow-up of the different lipid-ratios at 5 years follow-up using a comprehensive list of metabolic factors (listed in Table 3). Univariate and stepwise (probability of F to-enter 0.1-remove 0.15) linear regression analyses were carried out on log transformed variables (if skewed) and results given as standardized regression coefficients. Only variables below p < 0.2 were included in the stepwise multivariable models (the variables marked in bold). For determining the number of predictors we used the rule of thumb that we could include n ≥ 50 + 8 * number of predictors [28]. With, n = 272 we could include 25–30 independent predictors. Post-hoc power calculations revealed that the multiple regression models had power ~1.00. Two-tailed p values <0.05 were considered significant.

## Results

Table 1 shows the characteristics of the study population at the time of the 5-year follow-up visit stratified into those women who did and did not have GDM in the index pregnancy using WHO criteria (based on the OGTTs), which we have recently demonstrated are at particular CV risk (compared to IADPSG criteria) [27]. Women with GDM based on the WHO criteria had a higher BMI at week 14–16 [26.0 (22.6–28.7) kg/m^2^ vs. 23.6 (21.5–25.6) kg/m^2^] and at week 30–32 [27.8 (25.7–31.2) kg/m^2^ vs. 26.5 (24.0–28.6) kg/m^2^, p = 0.011 and p = 0.029 respectively], and as evident from Table 1, at follow-up after 5 years compared to their non-GDM counterparts and were more frequently smokers at follow-up. Further, the GDM women had a higher estimated insulin resistance, unfavorable lipid ratios (i.e., higher TG/HDL-C, LDL/HDL-C and apoB/apoA), lower estimated insulin sensitivity and lower estimated β-cell function at the 5-year follow-up visit.

**Table 1:** Characteristics of the study population at 5 year follow-up

	Non-GDM	GDM
N =	241	31
Follow-up time	4.8 (4.4, 5.4)	5.0 (4.5, 5.4)
Age (years)	37.5 ± 3.8	38.6 ± 3.8
Height (cm)	169 ± 6	168 ± 5
BMI (kg/m^2^)	22.8 (20.9, 25.2)	24.1 (21.7, 28.1)*
Primipara n (%)	217 (90.0)	25 (80.6)
Family history heart disease n	137 (57.3)	22 (75.9)
Family history diabetes n (%)	74 (30.7)	13 (41.9)
Currently smoking n (%)	38 (20.4)	9 (39.1)*
Previous smoker n (%)	55 (27.1)	8 (36.4)
Systolic blood pressure (mmHg)	110 (100, 120)	110 (100, 130)
Diastolic blood pressure (mmHg)	70 (60, 74)	70 (65, 80)
Insulin sensitivity	275 (196, 377)	210 (126, 304)**
Insulin resistance	0.67 (0.44, 0.97)	0.96 (0.48, 1.33)*
Β-cell function	1049 (816, 1373)	712 (559, 1247)**
TG/HDL-C ratio	0.46 (0.36, 0.63)	0.65 (0.45, 1.03)**
LDL/HDL-C ratio	1.65 (1.25, 2.04)	2.00 (1.62, 2.48)*
ApoB/ApoA ratio	0.44 (0.36, 0.53)	0.53 (0.42, 0.62)*

Data given as mean ± SD when normal distributed and median (25th, 75th) when skewed distributed. Preterm delivery (n = 12), hypertension (n = 7) and preeclampsia (n = 10) are excluded

* p < 0.05

** p < 0.001

### Women with GDM have higher plasma leptin, leptin/adiponectin ratio and lower adiponectin in pregnancy and at follow-up compared to non-GDM women

**Fig. 1:**
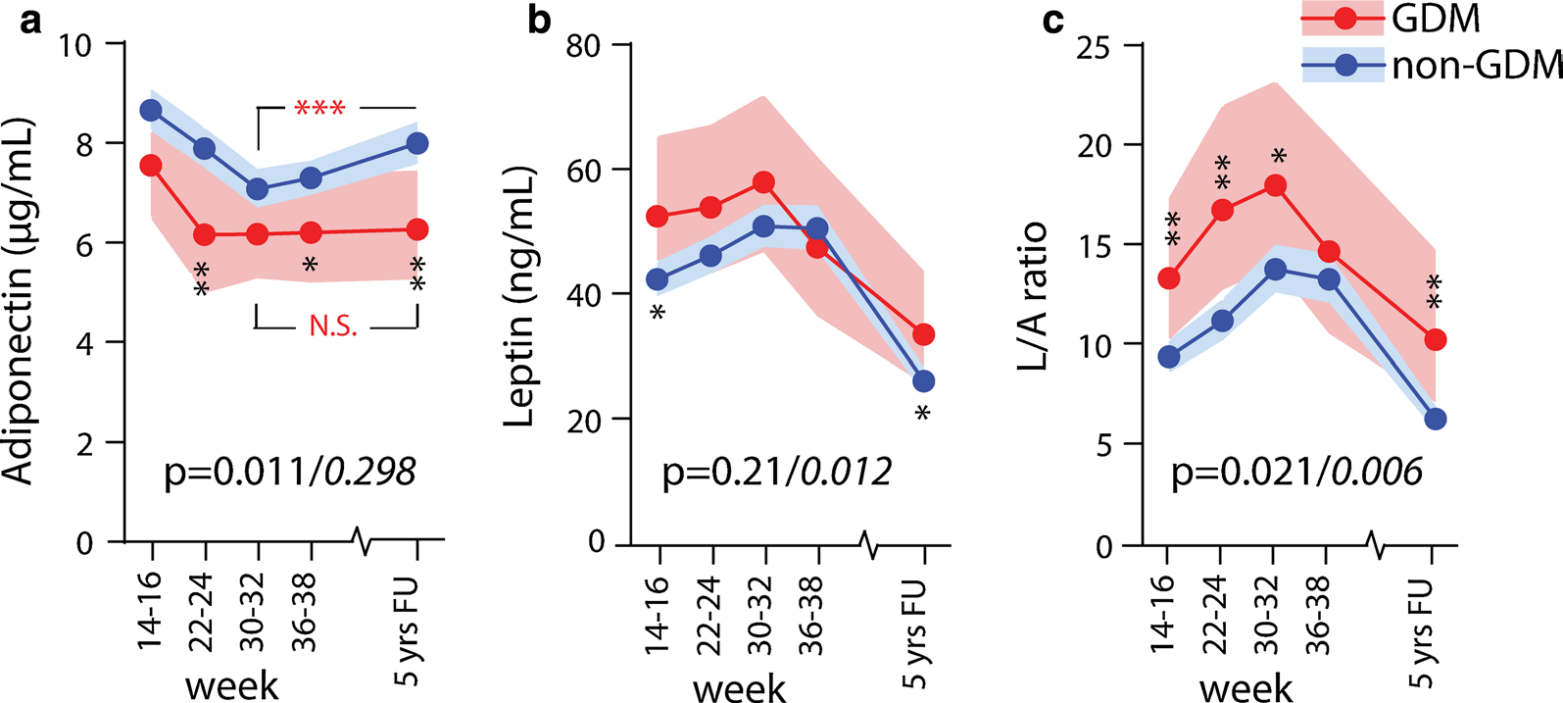
Circulating levels of **a** adiponectin, **b** leptin, and **c** L/A ratio in pregnancy and at 5-year follow-up between GDM (WHO) (*red line*) and non-GDM (*blue line*). FU, follow-up. Data is given as mean, 95% CI. *p < 0.05, **p < 0.01 GDM compared with non-GDM group. The p values in each graph represents the group effect/interaction term (in *italic*) from repeated measures ANOVA. Time was significant for all variables (p < 0.001)

As shown in Fig. 1a, plasma adiponectin decreased from 14–16 weeks to 30–32 weeks in non-GDM women and then increased to 5 years follow-up. In contrast, a marked decrease from 14–16 weeks to 22–24 weeks was observed in women with GDM, reaching levels significantly lower than in non-GDM women, and importantly, adiponectin remained low throughout the observation period showing significantly lower levels compared to non-GDM women after 5 years. Leptin levels increased during pregnancy from 14–16 weeks to 30–32 weeks with a marked decrease during follow-up in both GDM and non-GDM women, but notably, leptin levels were significantly higher at week 14–16 and 5 year follow-up in the GDM group (Fig. 1b). A similar temporal pattern as for leptin was found for the L/A ratio although women with GDM had significantly higher ratios at all time-points (except 36–38 weeks), Fig. 1c.

### Associations between L/A ratio and clinical characteristics in pregnancy

Our hypothesis was that the L/A ratio were associated with CV risk and we therefore evaluated the association between this ratio and clinical variables at each visit (Table 2). Briefly, BMI, systolic BP, CRP and estimated insulin resistance was consistently positively-, while estimated insulin sensitivity and β-cell function were consistently negatively, associated with the L/A ratio at each visit in univariate analysis. Stepwise multivariable linear regression revealed that BMI and estimated insulin sensitivity were the strongest predictors of the L/A ratio at most visits (Table 2).

**Table 2:** Association between L/A ratio and clinical characteristics throughout pregnancy and at 5 year follow-up

	14–16 weeks		22–24 weeks^c^		30–32 weeks		36–38 weeks^c^		5 year. follow-up	
	Uni	Multi	Uni	Multi	Uni	Multi	Uni	Multi	Uni	Multi
Age	−0.02		−0.01		−0.03		−0.13*	−0.12*	−0.01	
BMI	0.70***	0.53***	0.68***	0.50***	0.61***	0.38***	0.61***	0.50***	0.73***	0.56***
Parity^a^	0.05		0.02		−0.01		*−0.08*	−0.14**	0.04	
Smoking^b^	0.16*		0.15*		*0.09*		0.18**		0.01	
Systolic BP	0.26***		0.20**		0.22***		0.25***		0.28***	
CRP	0.25***		0.32***		0.28***		0.20***		0.32***	
Insulin sensitivity	−0.60***	−0.32***	−0.61**	−0.35***	−0.61***	−0.24*	−0.50***	−0.37***	−0.61***	−0.29***
Insulin resistance	0.56***		0.53***		0.59***	0.28*	0.48***		0.56***	
Β-cell function	−0.39***		−0.38***		−0.33***	0.20**	−0.18***	0.17**	−0.45***	
R-square		0.56		0.55				0.49		

Values from the previous visit were used. The independent variables with a p < 0.2 that is included in multivariable models are marked in italics

* p < 0.05

** p < 0.01

*** p < 0.001

^a^Primipara/multipara

^b^Previous and current, Linear regression (Beta, p)

^c^Measures of glucose metabolism not available at indicated time-point

### Leptin/adiponectin ratio in pregnancy as CV risk marker

**Fig. 2:**
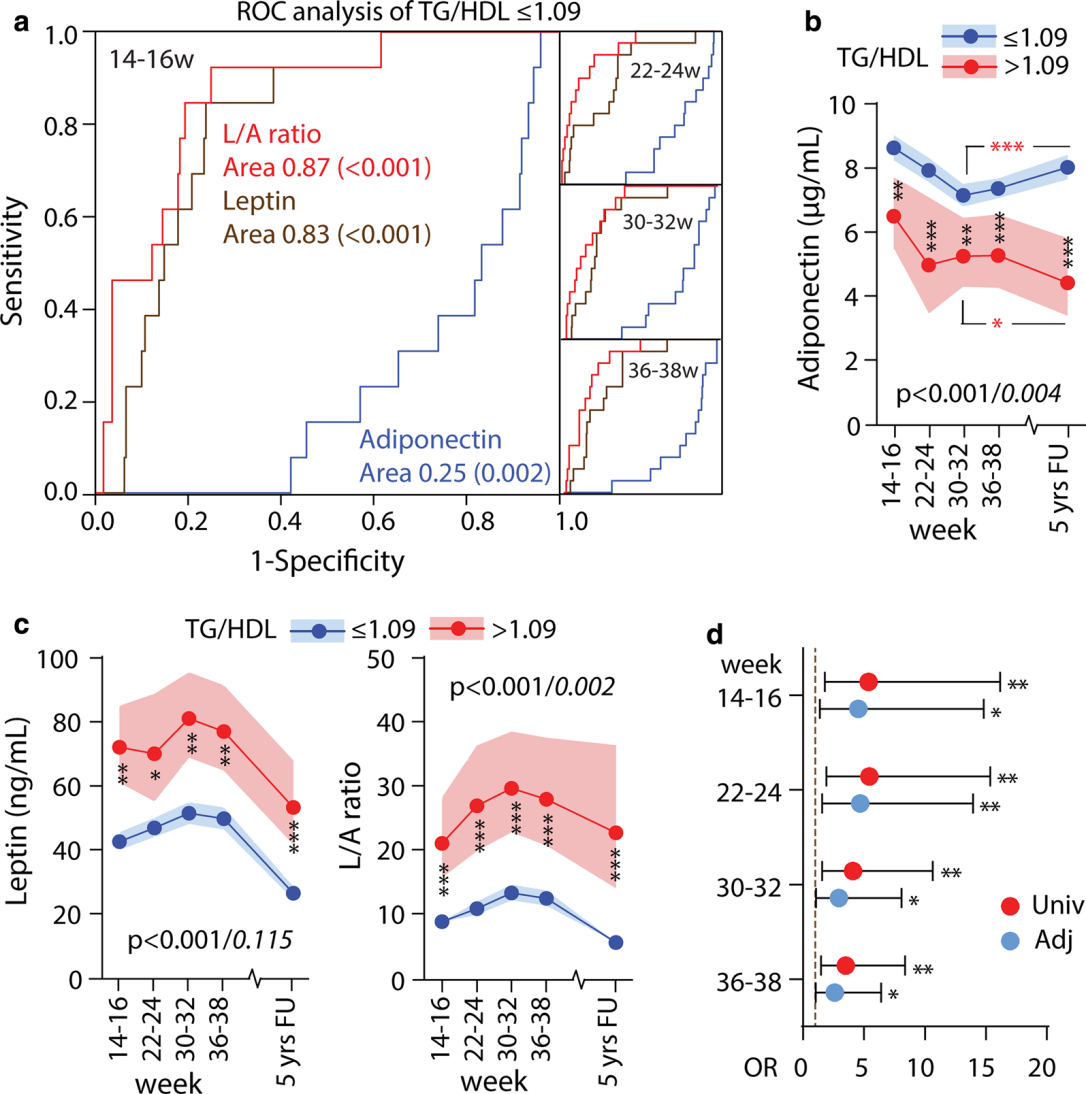
**a** Receiver operating characteristic (ROC) curves for predicting TG/HDL-C ratio at 5 year by adiponectin (*blue line*), leptin (*brown line*) and L/A ratio (*red line*) in pregnancy. **b** Adiponectin **c** leptin and L/A ratio in all pregnancy and 5 year follow-up divided by high (*red line*) and low (*blue line*) TG/HDL-C ratio. *p < 0.05, **p < 0. 01, ***p < 0.001. The p value represents the group effect/interaction term (in *italic*) from repeated measures ANOVA. Time was significant for all variables (p < 0.001). **d** Univariate (*red circles*) and adjusted (*blue circles*) models for increased CV risk as reflected by the TG/HDL-C ratio by L/A ratio during pregnancy. The adjusted analysis included BMI and estimated insulin sensitivity acquired at the same time as the L/A ratio

We have previously reported that GDM women in our study have enhanced CV risk based on elevated arterial stiffness and lipid ratios [27]. We next assessed if the L/A ratio at different time points in pregnancy could predict CV risk at 5 years follow-up based on TG/HDL-C ratio, apoB/apoA ratio and LDL/HDL-C ratio. ROC analysis (Additional file 1: Table S1) demonstrated that adiponectin, leptin and the L/A ratio significantly predicted enhanced CV risk based on established cut-offs [9, 21, 22] for the ratios of TG/HDL-C ratio, apoB/apoA ratio and LDL/HDL-C ratio at 5 years follow-up. The L/A ratio showed the highest AUCs for all lipid ratios, but the association with the TG/HDL-C ratio was particularly strong at all time-points (Fig. 2a). As shown in Fig. 2b and c, women with enhanced risk as suggested by increased TG/HDL-C (i.e. ≥1.09) at 5 years follow-up, displayed markedly lower adiponectin, and higher leptin and L/A ratios, at all time-points during and after pregnancy. However, in contrast to leptin, which displayed a similar pattern in the TG/HDL-C groups, adiponectin increased from week 30–32 to follow-up in women with low TG/HDL-C, but decreased in women with a high TG/HDL ratio (Fig. 2b).

Since BMI and estimated insulin sensitivity were strongly correlated with the L/A ratio, we evaluated if the association between the L/A ratio in pregnancy and enhanced CV risk as determined by a high TG/HDL-C ratio was modified by these measures using logistic regression. As shown in Fig. 2d, the OR’s were only slightly attenuated, and remained significant at all time-points after adjusting for BMI and estimated insulin sensitivity. A similar pattern, although of more modest statistical significance, was observed for the LDL/HDL-C and apoB/apoA ratios (Additional file 1: Table S2).

### L/A ratio and interaction with GDM on CVD risk

**Fig. 3:**
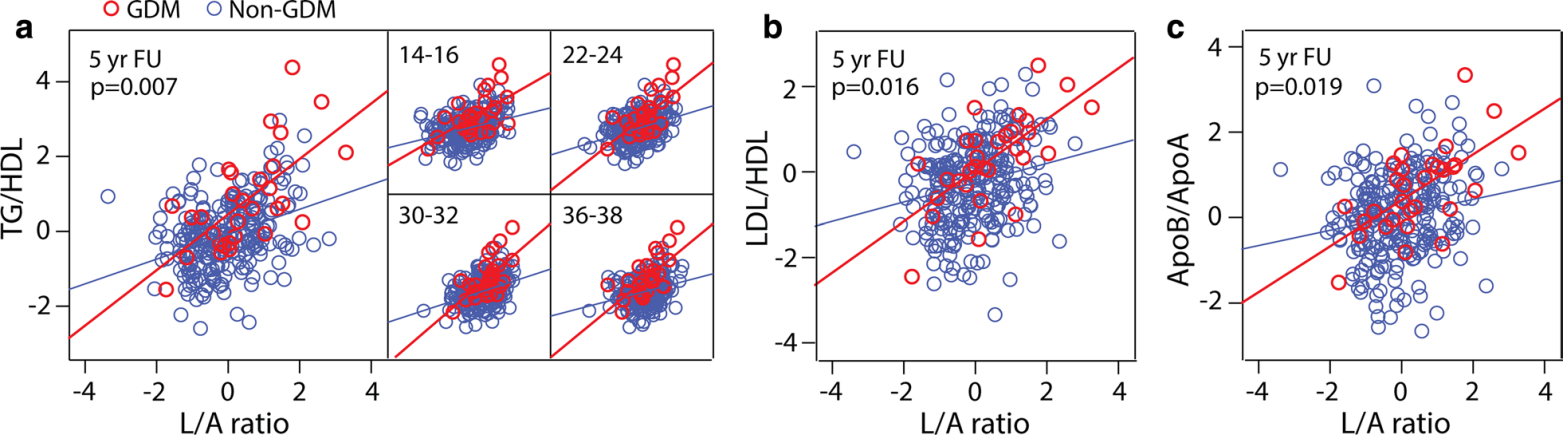
Interaction analysis between L/A ratio in pregnancy and 5-year follow-up and **a** TG/HDL-C ratio in GDM (*red circles*) and non-GDM (*blue circles*) women. Interaction analysis between L/A ratio at 5-year follow-up and **b** LDL/HDL-C ratio and **c** apoB/apoA ratio in GDM and non-GDM women. p values indicates interaction analysis

To further evaluate whether the associations between the L/A ratio and CV risk measurements were stronger in women with previous GDM, we performed an interaction analysis. As shown in Fig. 3a, we found a significant interaction between GDM and the L/A ratio at 5-year follow-up for the TG/HDL-C ratio, and this interaction was also present at all visits during pregnancy. Similar interactions between GDM and the L/A ratio were present for the LDL/HDL-C and apoB/apoA ratio at 5-year follow-up (Fig. 3b, c). Thus, an increase in the L/A ratio of 1 unit is associated with a larger increase in CV risk in women with previous GDM compared to uncomplicated pregnancy suggesting that a high ratio may enhance the adverse effects of glucose intolerance on the TG/HDL ratio. The change in the L/A ratio from visit 1 to 4 in pregnancy was associated with the TG/HDL-C ratio at follow-up in GDM women (r = 0.49, p = 0.008) but no associations was observed in controls (r = 0.01, p = 0.90).

### Predictors of cardio-metabolic risk factors 5-year follow-up

We finally evaluated the association between adiponectin, leptin, the L/A ratio and lipid ratios as continuous variables at 5-years follow-up in regression models with established CVD risk factors. As shown in Table 3, despite slightly weaker or similar correlations with the lipid ratios as the L/A ratio, adiponectin together with estimated insulin sensitivity or insulin resistance, remained a predictor in the final model, suggesting effects on CV risk independent of associations with glucose metabolism. PWV (reported previously in [27] correlated modestly with leptin (r = 0.14, p = 0.022), the L/A ratio (r = 0.12, p = 0.041), but not with adiponectin (r = −0.03, p = 0.59) and was also a predictor in the final model for apoB/apoA ratio. Smoking, systolic BP, CRP and GDM were included as predictors in the final model when investigating TG/HDL-C ratio.

**Table 3:** Markers of cardio-metabolic risk factors at 5-year follow-up for the whole cohort

	TG/HDL ratio		ApoB/apoA ratio		LDL/HDL ratio	
	Uni	Multi	Uni	Multi	Uni	Multi
Follow-up	0.01		−0.01		−0.04	
Age	0.02		−0.01		0.01	
BMI	0.41***		0.28***		0.31***	
Parity	−0.06		*−0.09*		*−0.08*	
Smoking	0.14*	0.13*	0.07		0.04	
Diabetes in family	*0.10*		*0.09*		0.07	
Heart disease in family	0.03		0.01		0.04	
Systolic BP	0.27***	0.22**	*0.10*		*0.11*	
Diastolic BP	0.13*		0.02		0.01	
CRP	0.30**	0.10	*0.13*		0.15*	
Insulin sensitivity	−0.50***	−0.31***	−0.33***	−0.25***	−0.39***	
Insulin resistance	0.44***		0.31***		0.36***	0.32***
Β-cell function	−0.40***		−0.29***		−0.32***	
PWV	0.24***		0.15*	0.10	0.15*	
Visceral fat	0.41***		0.30***		0.32***	
Leptin	0.32***		0.16**		0.21***	
Adiponectin	−0.37***	−0.18**	−0.26***	−0.17**	−0.27***	0.17**
L/A ratio	0.43***		0.25 ***		0.31***	
GDM WHO	0.27**	0.16**	0.18**		0.18**	
R square		0.35		0.15		0.17

The independent variables with a p < 0.2 that is included in multivariable models are marked in italics

* p < 0.05

** p < 0.01

*** p < 0.001

^a^Primipara/multipara

^b^Previous and current, Linear regression (Beta, p)

## Discussion

The present study investigated the significance of leptin, adiponectin and their ratio in pregnancy and follow-up between GDM vs. non-GDM women and associations with CV risk as evaluated by unfavorable lipid ratios at 5 years follow-up. Our main findings were: (1) The L/A ratio was higher in GDM women both during pregnancy and follow-up compared to non-GDM women (2) a high L/A ratio during pregnancy was associated with CV risk based on unfavorable lipid ratios at follow-up, especially the TG/HDL-C ratio (3) interaction analysis indicated that an increase in the L/A ratio of 1 unit was associated with a higher CV risk in GDM compared to normal pregnancy (4) low adiponectin levels independently predicted unfavorable lipid ratios at follow-up. Our findings suggest that high L/A ratio is associated with CV risk in women with GDM, potentially also being involved in the pathogenesis of CVD in these women.

Our study demonstrates that circulating adiponectin levels are lower early in pregnancy in women who develop GDM supporting a number of studies evaluating this protein during pregnancy as highlighted in a recent meta-analysis [29]. Conversely, increased leptin levels have been reported frequently in GDM [16] although this increase was less impressive in our study. This could be a power issue, although Retnakaran et al. found no difference in 2nd trimester leptin levels when evaluating the impact of glucose tolerance in pregnancy (n = 487) including 137 GDM women [17]. Indeed, experimental studies indicate that the effects of leptin on glucose homeostasis are suppressed during pregnancy [30]. Furthermore, both diet and low birthweight may have an impact on leptin levels, irrespective of body mass [31, 32]. A low birth weight was associated with a higher prevalence of diabetes and obesity, higher leptin levels and leptin to fat mass ratio possibly related to nutrition or the development of leptin resistance and/or higher leptin production by body fat unit [32]. Fewer studies have evaluated the longitudinal trajectory of these proteins during pregnancy and beyond. In contrast to normal pregnancy, where the lowest adiponectin levels were observed at 30–32 weeks and then increased steadily, adiponectin floored at 22–24 weeks in GDM women and remained low also at the 5 year follow-up. Thus, our data extend previous findings by showing that persistently low adiponectin levels, also during follow-up after pregnancy may represent a risk factor for future development of metabolic and CV disease in GDM women.

We and others have shown that women with a history of GDM display an unfavorable lipid profile [27, 33, 34] with enhanced ratios such as TG/HDL-C as a marker of cardiometabolic risk [27]. While leptin and adiponectin, and their association with measures of adiposity and glucose metabolism are well established in GDM, their associations with surrogate markers of CV risk in these women, or during normal pregnancy, are less documented. Men with a high ratio of adiponectin/leptin (opposite of L/A ratio) had lower TG and TG/HDL-C ratios [35]. It has been suggested that the L/A ratio may serve as a potential atherogenic index in obese T2DM patients [11] and in metabolic syndrome [36]. A close correlation with carotid intima-media thickness, a surrogate marker of atherosclerosis, has been documented in T2DM [10] and in healthy subjects [37]. We have previously demonstrated a higher arterial stiffness as measured by PWV at 5 years follow-up in these GDM women [27], but detected only a modest association between leptin and the L/A ratio and PWV in the present study. Thus, our findings are limited by the relatively young age of the women and lack of manifest CVD during follow-up. Nonetheless, our finding that the L/A ratio throughout pregnancy consistently predicted CV risk at 5 years follow-up, better than adiponectin or leptin alone, and with good accuracy based on the apoB/apoA ratio, LDL/HDL-C ratios, and in particular the TG/HDL-C ratio, suggest that a dysregulated leptin and adiponectin profile should be further evaluated in relation to CVD during long-term follow-up after pregnancy.

Previous studies have demonstrated an association between the L/A ratio, BMI and insulin resistance in obese patients, patients with metabolic syndrome and in non-diabetic adults [38, 39]. Similar associations were also observed in pregnant women [18] and our finding that BMI and estimated insulin sensitivity were major predictors of the L/A ratio throughout pregnancy support these findings. Importantly, however, the association between the L/A ratio and an elevated TG/HDL-C ratio at 5 years persisted following adjustment for these factors, with similar, albeit weaker, associations with the other lipid ratios especially towards the end of pregnancy. In addition to contributing to enhanced CV risk through metabolic pathways related to adiposity and insulin resistance, adiponectin and/or leptin could therefore have independent effects as well [11]. Our finding that adiponectin, but not the L/A ratio, was a predictor of the TG/HDL-C ratio at 5 years follow-up, may suggest that while the L/A ratio represents a stronger risk marker, this risk is at least partly mediated by adiponectin. Indeed, adiponectin may directly affect the metabolism of HDL and TG rich lipoproteins [40, 41]. Furthermore, adiponectin attenuates the inflammatory response to multiple stimuli by modulating signaling pathways in a variety of cell types [42]. Both adiponectin and leptin have been shown to affect atherosclerotic progression [43]. While adiponectin may be protective in atherosclerosis by diminishing endothelial injury, which is the first step in atheroma formation [44], leptin increase the secretion of inflammatory markers [45]. Adiponectin may also inhibit endothelial inflammation and correlates negatively with vascular inflammation markers such as VCAM-1 and ICAM-I [46]. Conversely, leptin increases these markers, which favors monocyte attraction and migration through the endothelial wall [47]. Adiponectin may also modulate macrophages to an anti-inflammatory phenotype and inhibit foam cell transformation [48]. Thus, having a dysregulated adiponectin and leptin profile, as showed in GDM women, may potentially have deleterious effects on atherosclerosis development.

In addition to be a risk marker for CVD through its effect on L/A ratio, adiponectin could also be involved in the pathogenesis of CVD following GDM. Finally, our interaction analysis showing that a change in the L/A ratio throughout pregnancy and at follow-up was associated with a larger increase in the TG/HDL-C, as well as the LDL/HDL-C and apoB/apoA ratio, supports that the L/A ratio may be of particular relevance in GDM and enhance the adverse effects of glucose intolerance on CV risk. Indeed, while change in the L/A ratio from early to late pregnancy was not associated with any of the lipid ratios in normal pregnancy, this change correlated well with the TG/HDL-C ratio in GDM women.

## Conclusions

Taken together, our findings suggest that high L/A ratio in pregnancy and in particularly in those with GDM are associated with an unfavorable CV risk profile during follow-up. Future studies should investigate if a dysregulated leptin and adiponectin profile during pregnancy is associated with atherosclerotic disease during long-term follow-up.

## Additional file

**Additional file 1: Table S1.** ROC analysis demonstrated that adiponectin, leptin and the L/A ratio significantly predicted enhanced CV risk based on established cut-offs for the ratios of TG/HDL-C ratio, apoB/apoA ratio and LDL/HDL-C ratio at 5 years follow-up. **Table S2**. Logistic regression analysis showing Odds Ratio and 95% CI for the prediction of increased lipid ratios by the L/A ratio at different timepoints during pregnancy.

## Abbreviations

Apo: apolipoprotein
CVD: cardiovascular disease
GDM: gestational diabetes mellitus
HDL-C: high density lipoprotein cholesterol
HOMA-IR: homeostasis model assessment-insulin resistance
IL: interleukin
L/A: leptin/adiponectin
LDL-C: low density lipoprotein cholesterol
OGTT: oral glucose tolerance test
PWV: pulse wave analysis
T2DM: type 2 diabetes patients
TG: triglycerides
VAT: visceral adipose tissue
