# Supplementary material for: Leptin and adiponectin as predictors of cardiovascular risk after gestational diabetes mellitus
Source: Cardiovasc Diabetol. 2017 Jan 10;16:5. doi: 10.1186/s12933-016-0492-4 (PMC5223461; doi:10.1186/s12933-016-0492-4)
Supplement: Supplementary file 1 — Additional file 1: Table S1. ROC analysis demonstrated that adiponectin, leptin and the L/A ratio significantly predicted enhanced CV risk based on established cut-offs for the ratios of TG/HDL-C ratio, apoB/apoA ratio and LDL/HDL-C ratio at 5 years follow-up. Table S2. Logistic regression analysis showing Odds Ratio and 95% CI for the prediction of increased lipid ratios by the L/A ratio at different timepoints during pregnancy. [file 12933_2016_492_MOESM1_ESM.docx]

Supplemental Table 1.

ROC analysis demonstrated that adiponectin, leptin and the L/A ratio significantly predicted enhanced CV risk based on established cut-offs for the ratios of TG/HDL-C ratio, apoB/apoA ratio and LDL/HDL-C ratio at 5 years follow-up.

|  |  | 14-16 weeks | 22-24 weeks | 30-32 weeks | 36-38 weeks |
| --- | --- | --- | --- | --- | --- |
| TG/HDL | Adiponectin | 0.25 (0.15-0.37)** | 0.24 (0.12-0.36)** | 0.27 (0.14-0.39)** | 0.23 (0.12-0.34)** |
|  | Leptin | 0.83 (0.74-0.91)*** | 0.75 (0.62-0.87)** | 0.80 (0.71-0.89)*** | 0.77 (0.68-0.87)** |
|  | L/A ratio | 0.87 (0.79-0.96)*** | 0.87 (0.79-0.94)*** | 0.86 (0.79-0.92)*** | 0.86 (0.78-0.93)*** |
|  |  |  |  |  |  |
| ApoB/ApoA | Adiponectin | 0.25 (0.11-0.37)* | 0.24 (0.09-0.38)* | 0.21 (0.09-0.37)* | 0.20 (0.09-0.32)** |
|  | Leptin | 0.79 (0.72-0.85)** | 0.73 (0.58-0.88)* | 0.72 (0.60-0.84)* | 0.77 (0.59-0.96)* |
|  | L/A ratio | 0.85 (0.76-0.93)** | 0.81 (0.67-0.96)** | 0.83 (0.68-0.98)** | 0.84 (0.67-1.00)** |
|  |  |  |  |  |  |
| LDL/HDL | Adiponectin | 0.28 (0.16-0.40)* | 0.30 (0.16-0.40)* | 0.30 (0.13-0.47)* | 0.27 (0.14-0.39)** |
|  | Leptin | 0.76 (0.64-0.89)** | 0.64 (0.48-0.81) | 0.72 (0.60-0.84)* | 0.70 (0.54-0.86)* |
|  | L/A ratio | 0.81 (0.69-0.93)** | 0.76 (0.62-0.90)** | 0.78 (0.65-0.91)** | 0.77 (0.61-0.93)** |

p<0.05 *, p<0.01**, p<0.001***

Supplemental Table 2.

Logistic regression analysis showing Odds Ratio and 95% CI for the prediction of increased lipid ratios by the Leptin/adiponection ratio at different timepoints during pregnancy

|  | TG/HDL-C>1.09 | | LDL/HDL-C>3.0 | | apoB/apoA>0.79 | |
| --- | --- | --- | --- | --- | --- | --- |
| L/A ratio | Uni | Adj. | Uni | Adj. | Uni | Adj. |
| 14-16 weeks | 6.18 (2.64-14.45)** | 4.54 (1.39-14.83)* | 3.77 (1.69-8.45)* | 4.54 (1.35-15.25)* | 4.46 (1.58-12.63)* | 4.96 (1.15-21.39)* |
| 22-24 weeks | 6.21 (2.63-14.67)** | 4.68 (1.58-13.93)** | 3.38 (1.53-7.45)** | 3.30 (1.13-9.65)* | 4.36 (1.53-12.41)* | 4.00 (1.08-14.77)* |
| 30-32 weeks | 5.34 (2.36-12.08)** | 2.94 (1.07-8.09)* | 3.76 (1.66-8.52)* | 2.55 (0.89-7.28) | 5.02 (1.69-14.91)* | 2.84 (0.78-10.38) |
| 36-38 weeks | 4.75 (2.24-10.05)** | 2.59 (1.05-6.40)* | 3.50 (1.64-7.49)* | 2.27 (0.89-5.82) | 5.13 (1.90-13.82)* | 2.99 (0.94-9.50) |

Model 1: univariate, Model 2: adjusted for BMI and insulin sensitivity, p<0.05 *, p<0.001**
